# Low Magnesium Levels and FGF-23 Dysregulation Predict Mitral Valve Calcification as well as Intima Media Thickness in Predialysis Diabetic Patients

**DOI:** 10.1155/2015/308190

**Published:** 2015-05-18

**Authors:** Ana Paula Silva, Kristina Gundlach, Janine Büchel, Teresa Jerónimo, André Fragoso, Claudia Silva, Patrícia Guilherme, Nélio Santos, Marília Faísca, Pedro Neves

**Affiliations:** ^1^Nephrology, Hospital de Faro, Rua Leão Penedo, 8000-386 Faro, Portugal; ^2^Fresenius Medical Care Deutschland GmbH, 61352 Bad Homburg, Germany; ^3^Pathology Clinic, Hospital de Faro, 8000-386 Faro, Portugal; ^4^Cardiology, Hospital de Faro, 8000-386 Faro, Portugal; ^5^Pharmacology, Gnostic Laboratory, 8000-386 Faro, Portugal

## Abstract

*Background*. Mitral valve calcification and intima media thickness (IMT) are common complications of chronic kidney disease (CKD) implicated with high cardiovascular mortality. *Objective*. To investigate the implication of magnesium and fibroblast growth factor-23 (FGF-23) levels with mitral valve calcification and IMT in CKD diabetic patients. *Methods*. Observational, prospective study involving 150 diabetic patients with mild to moderate CKD, divided according to Wilkins Score. Carotid-echodoppler and transthoracic echocardiography were used to assess calcification. Statistical tests used to establish comparisons between groups, to identify risk factors, and to establish cut-off points for prediction of mitral valve calcification. *Results*. FGF-23 values continually increased with higher values for both IMT and calcification whereas the opposite trend was observed for magnesium. FGF-23 and magnesium were found to independently predict mitral valve calcification and IMT (*P* < 0.05). Using Kaplan-Meier analysis, the number of deaths was higher in patients with lower magnesium levels and poorer Wilkins score. The mean cut-off value for FGF-23 was 117 RU/mL and for magnesium 1.7 mg/dL. *Conclusions*. Hypomagnesemia and high FGF-23 levels are independent predictors of mitral valve calcification and IMT and are risk factors for cardiovascular mortality in this population. They might be used as diagnostic/therapeutic targets in order to better manage the high cardiovascular risk in CKD patients.

## 1. Introduction

Patients with chronic kidney disease (CKD) are particularly susceptible to cardiovascular complications, and cardiovascular disease accounts for more than 50% of all deaths in this population [1]. Common complications like vascular (VC) and mitral valve calcification are clinically observed as changes in intima media thickness (IMT), coronary artery calcification, pulse pressure, or pulse wave velocity and have been implicated with the high cardiovascular mortality incidences observed [2, 3].

It is generally accepted that the interplay between kidney, bone, and vessels is important for maintaining mineral and bone homeostasis. It is during the early stages of renal disease that mechanisms responsible for keeping the balance start to get out of control, and an imbalance between inhibitory and inducing mediators has been shown to be the driving force for VC and mitral valve calcification [4]. Thus, especially the earlier stages of renal failure might be critical for the onset of the calcification process and for potential therapeutic interventions [5].

For many years calcification was believed to be an inert process, resulting exclusively from elevated concentrations of serum phosphate and calcium phosphate product [6–8]; however, it is currently being understood as a multifactorial, active, and dynamic process that shares similarities with osteogenic differentiation [9]. Several factors are known to be involved in triggering the calcification process and inducing the phenotypical transformation of vascular smooth muscle to bone-forming cells [10].

Magnesium could be one potentially important factor in this process as several recent studies in the general population, in predialysis CKD patients, and in hemodialysis patients have shown a correlation of low magnesium levels with all-cause and cardiovascular mortality [11–14]. Low serum magnesium levels have been further associated with VC, both in animals [15–17] as well as in clinical observational [18–21] and interventional studies [22, 23] in hemodialysis patients. Last but not least low serum magnesium levels have also been associated with higher IMT values in several studies with dialysis or hypertensive patients [23–25].

Another important factor is fibroblast growth factor-23 (FGF-23). FGF-23 levels, known to rise early in patients with CKD [26], have recently been implicated with VC, left ventricular hypertrophy, endothelial dysfunction, and increased mortality in dialyzed patients [27, 28].

Therefore, both serum magnesium and FGF-23 seem to be potential markers of valvular calcification [29]. However, and to our knowledge, no study so far has assessed these two variables conjointly in a population of diabetics with impaired kidney function. Nonetheless, such analyses are of value as they provide insights into the early development of CKD and associated cardiovascular complications.

In the present study we investigated the association of serum levels of magnesium and FGF-23 with mitral valve calcification and IMT in diabetic patients with mild to moderate CKD to further elucidate the clinical developments for possible therapeutic approaches.

## 2. Material and Methods

This is a prospective, observational study in diabetic patients with mild to moderate CKD. Patients were screened and recruited in an outpatient diabetic nephropathy clinic and were followed from January 2008 to December 2013. Before its implementation, the study was submitted and approved by the local Ethics Committee. All principles of the Declaration of Helsinki of 1975, as revised in 2000, were followed and study procedures were only conducted after obtaining patients' written informed consent.

### 2.1. Subjects

In this study 150 patients with type 2 diabetes and with mild to moderate CKD (15 mL/min/1.73 m^2^ < eGFR ≤ 89 mL/min/1.73 m^2^) were included. The classification of diabetes followed the guidelines established by the American Diabetes Association [30]. All included patients were, at the time of inclusion, undergoing several pharmacologic therapies, namely: antihypertensive drugs such as antagonist of receptor of angiotensin (ARA) and angiotensin converting enzyme inhibitor (ACEI), antidyslipidemic drugs, acetylsalicylic acid (ASA), and oral antidiabetic agents.

Patients were considered ineligible to participate in the study if they presented at least one of the following criteria: previous cardiovascular disease (defined as a history of one or more of the following: nonfatal myocardial infarction, angina pectoris (stable or unstable), stroke or transient ischemic attacks, and congestive heart failure), history of valvulopathies (including rheumatic fever), uncontrolled hypertension (BP ≥ 140/90 mmHg), albumin/creatinine ratio (UACR) > 500, estimated glomerular filtration rate (eGFR) ≤ 15 mL/min or ≥90 mL/min, parathyroid hormone (PTH) ≥ 350 pg/mL, phosphorus > 5.5 mg/dL, type 1 diabetes, renal disease other than diabetic nephropathy, and neoplastic or infectious diseases. Patients were not allowed to undergo therapy with thiazide or loop diuretics, spironolactone, magnesium supplements, or any laxative or chelate agent containing magnesium. Patients with any gastrointestinal pathology that could possibly interfere with magnesium absorption were also not included in this study.

All mortalities caused by other than cardiovascular events were also excluded.

### 2.2. Followup

Followup of patients was conducted 2-3 times a year during in-person visits on nephrology consultation. Patients with more severe conditions returned approximately every 3 months, with the other patients returning every 6 months. No patient was “lost to followup” since in the Algarve region all patients with renal disease are referred to Hospital de Faro, with the continuity of the followup being assured.

### 2.3. Blood Measurements

Serum samples were collected at baseline in fasting patients. Samples were centrifuged and plasma was frozen at −80°C. Several laboratory parameters were analyzed: glycated hemoglobin (HbA1c), lipid profile [total cholesterol, high-density lipoprotein (HDL) cholesterol, low-density lipoprotein (LDL) cholesterol, and triglycerides], mineral metabolism [calcium, phosphorus, magnesium, PTH], inflammation [interleukin-6 (IL-6)], active form of vitamin D [1,25(OH)_2_D3], FGF-23, and serum creatinine.

FGF-23 serum levels were quantified using an enzyme-linked immunosorbent assay,* Human FGF-23 (C-Term)* ELISA kit (Cat. #60-6100 Immutopics Inc., San Clemente, CA, USA). Serum levels of 1,25(OH)_2_D3 were quantified with a radioimmunoassay (IDS, Boldon, UK). Total cholesterol, HDL, phosphorus, and magnesium were measured using the ARCHITECT c Systems and the AEROSET System (Abbott Diagnostics Division, Abbott Laboratories Abbott Park, IL, USA) and LDL cholesterol in human plasma was assessed using a MULTIGENT Direct LDL assay (Abbott Diagnostics Division, Abbott laboratories Abbott Park, IL, USA). Serum levels of IL-6 were measured using a sandwich enzyme-linked immunoassay (ELISA) kit (eBioscience, San Diego, CA, USA). HbA1c and PTH levels were measured using a spectrophotometry technique and electrochemiluminescent immunoassays (ECLIA), respectively.

### 2.4. Renal Function Assessment

Values of serum creatinine were obtained through an enzymatic method, using the ARCHITECT device (Abbott Diagnostics Division, Abbott Laboratories Abbott Park, IL, USA), while GFR was estimated using a formula derived by the Modification of Diet in Renal Disease study group [31].

### 2.5. Echocardiography

Transthoracic echocardiography was performed using a General Electrical Medical Systems echograph, model Vivid 7 with a probe (GE Healthcare, WI, USA). Data were recorded on computer and film and were always analyzed by the same technician.

### 2.6. Carotid Echodoppler

Carotid echodoppler was performed using a General Electrical Medical Systems echograph, model Vivid 4 with a linear probe of 10 MHz (GE Healthcare, WI, USA). For the assessment of the carotid artery intima-media thickness (IMT), the protocol of the American Society of Echocardiography was followed [32]. Data were recorded and analyzed by the same technician.

### 2.7. Outcomes

The primary outcome event studied was the presence of calcifications on the mitral valve annulus. The presence and extent of calcifications were assessed through echocardiographic examinations. Depending on the features of the echocardiographic findings, patients were stratified and divided into 4 groups according to the extent of mitral valve calcifications. This grading was performed following the Wilkins score, modified by Soliman and colleagues (Table 1) [33].

### 2.8. Statistical Analyses

Analyses were performed by using descriptive statistics, and for comparisons between groups ANOVA with Scheffé post hoc tests were used. Survival was estimated with the Kaplan-Meier method and the comparison between groups was made by using the log-rank test. Multivariate linear regressions were applied in order to identify risk factors. Receiver operating characteristic (ROC) curves were drawn in order to analyze sensitivity and specificity and to determine a cut-off point for serum FGF-23 and magnesium levels for predicting mitral valve calcification. In all analyses, *P* < 0.05 was considered significant. All analyses were performed using the SPSS program, v17.0.

## 3. Results

Patients' baseline characteristics are summarized in Table 2. The mean age of the patients was 66.6 ± 9.7 years (40–85) and 35.3% (53) were female. The study was conducted for 72 months, between January 2008 and December 2013.

**Figure 1:**
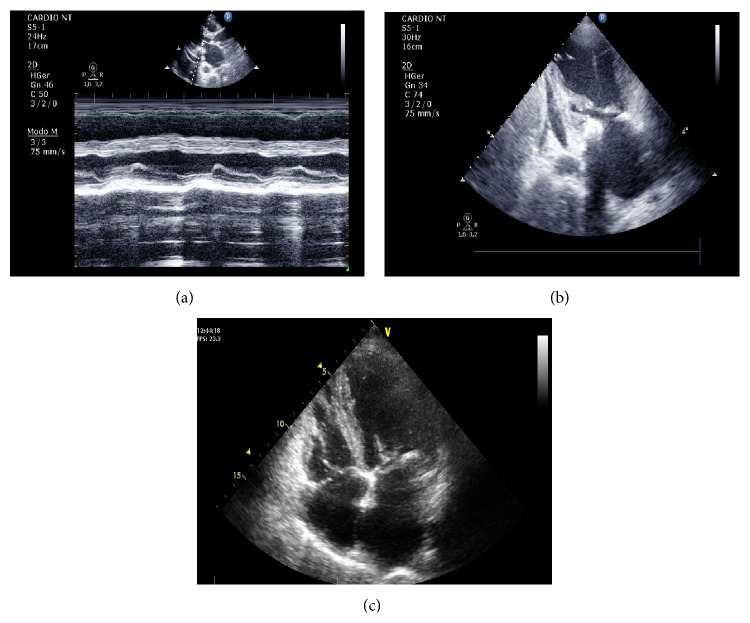
Echocardiographic findings: (a) parasternal incidence long axis, mitral valve M-mode, fibrotic leaflets, with limited leaflet excursion mobility, and annular calcification; (b) apical 2-chamber view, left LV, LA, mitral valve with fibrotic leaflet, and subvalvular apparatus and annular calcification; (c) apical 4-chamber view, LV, LA, RV, RA, mitral valve with fibrotic leaflet, and subvalvular apparatus and annular calcification.

**Figure 2:**
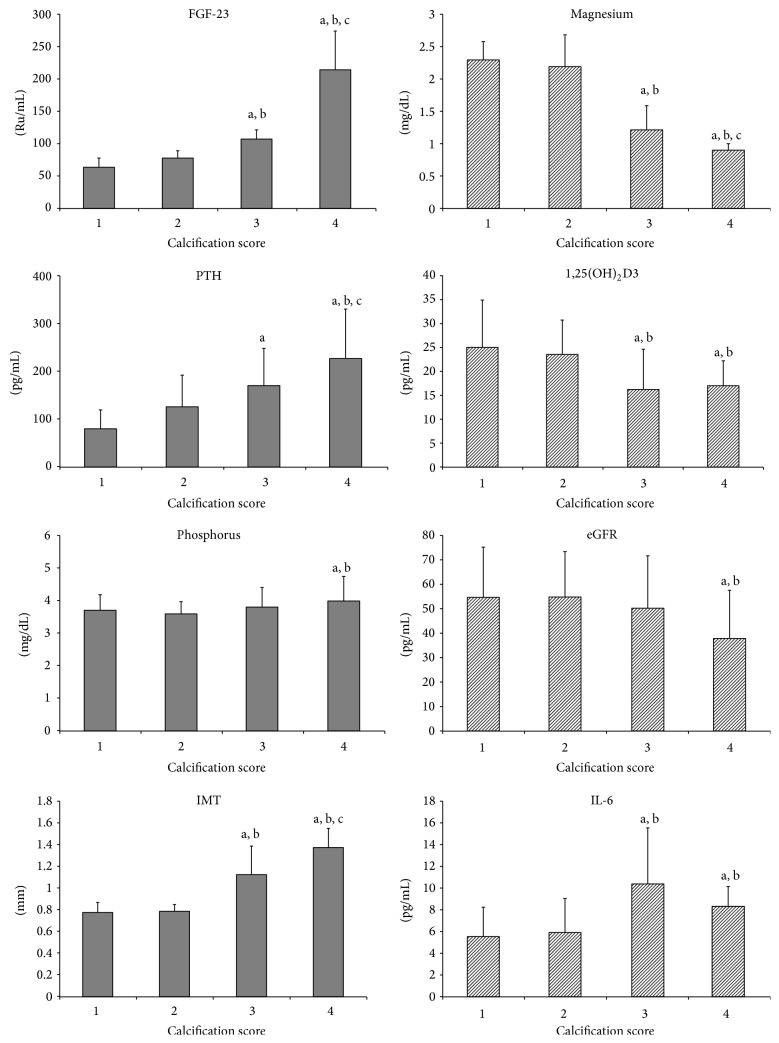
Parameters according to calcification score, 1 (*n* = 38), 2 (*n* = 47), 3 (*n* = 29), and 4 (*n* = 36). Results of post hoc analysis: a—*P* < 0.05 versus Group 1, b—*P* < 0.05 versus Group 2, and c—*P* < 0.05 versus Group 3.

After the echocardiographic assessments (Figure 1) patients were divided into 4 groups according to their Wilkins scores, 38 patients were allocated to Grade 1, 47 to Grade 2, 29 to Grade 3, and 36 patients to Grade 4. All the parameters assessed and depicted in Figure 2 present statistically significant differences between calcification groups.

Patients with poorer calcification features (Grade 4) presented higher levels of phosphorus, PTH, and FGF-23, as well as lower values of eGFR and magnesium. Continuous increase, accompanied by higher grading score, was seen for creatinine, PTH, and FGF-23 levels, whereas a continuous decrease in eGFR and magnesium levels was observed (Figure 2).

Variables such as age, calcium, phosphorus, Ca x P, PTH, creatinine, eGFR, FGF-23, magnesium, 1,25(OH)_2_D3, and IL-6 were analyzed using a multivariate linear regression to identify independent risk factors of mitral valve calcification. Calcium, eGFR, FGF-23, and magnesium were found to independently predict mitral valve calcification (*P* < 0.05) in opposition to the other variables (Table 3).

**Figure 3:**
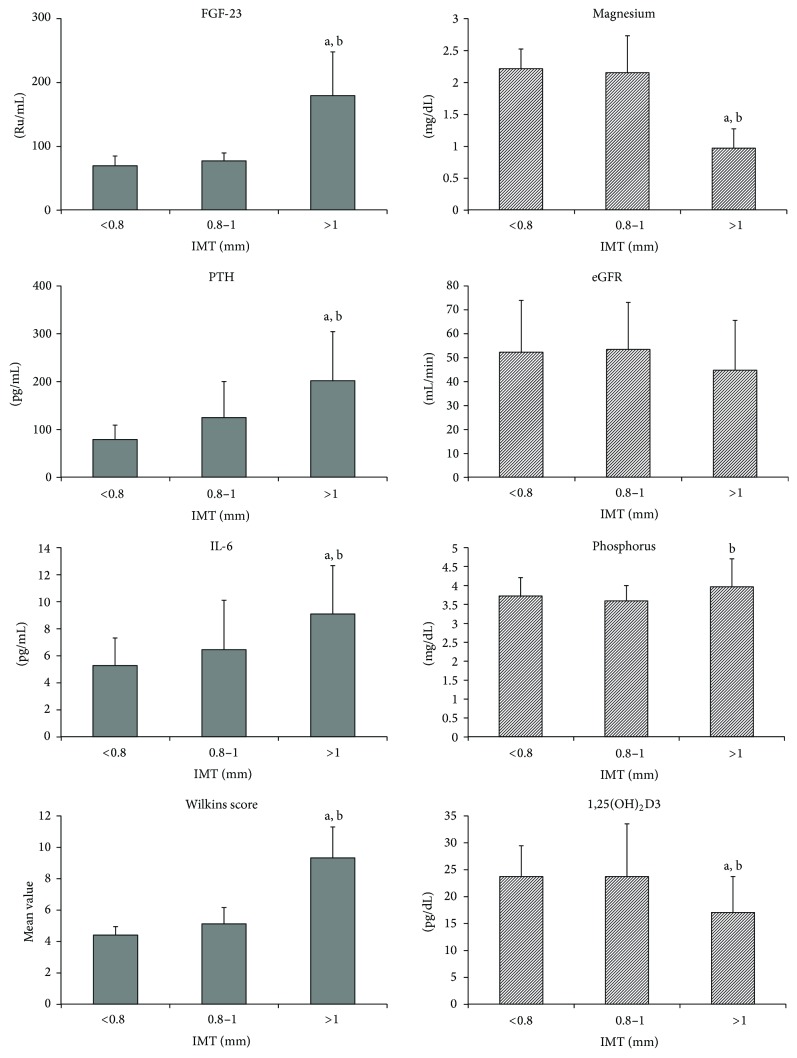
Parameters according to IMT values, <0.8 mm (*n* = 25), 0.8–1 mm (*n* = 69), and >1 mm (*n* = 56). Results of post hoc analysis: a—*P* < 0.05 versus Group 1 and b—*P* < 0.05 versus Group 2.

The trend of variables behavior was also assessed for IMT levels. Three groups were defined according to the IMT levels < 0.8, 0.8–1, and >1 mm and were analysed. Results demonstrated that variables such as FGF-23, PTH, and IL-6 continually increased with higher IMT levels, and magnesium and 1,25(OH)_2_D3 presented an opposite trend (Figure 3).

Furthermore, all variables were analyzed to identify independent risk factors of carotid intima-media thickness (IMT). IL-6, Wilkins score, FGF-23, and magnesium were found to independently predict IMT (*P* < 0.05) in contrast to the other variables (Table 4).

**Figure 4:**
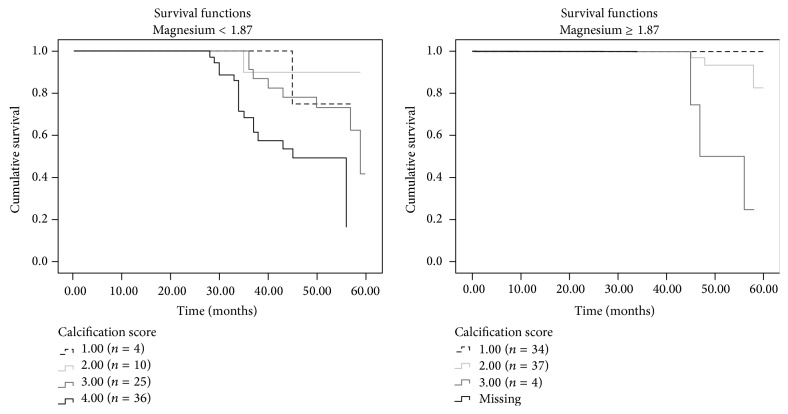
Survival analysis according to the serum magnesium level [mg/dL].

For detailed analyses all patients were grouped according to their magnesium levels resulting in 75 patients with levels lower than 1.85 mg/dL (= 0.76 mmol/L) (calcification score 1: *n* = 4; 2: *n* = 10; 3: *n* = 25; 4: *n* = 36) and 75 patients with levels of 1.85 mg/dL or higher (calcification score 1: *n* = 34; 2: *n* = 37; 3: *n* = 4; 4: *n* = 0). Using Kaplan-Meier analysis it was observed that the number of deaths was higher in patients with lower magnesium levels as well as in patients with poorer Wilkins score (Figure 4).

**Figure 5:**
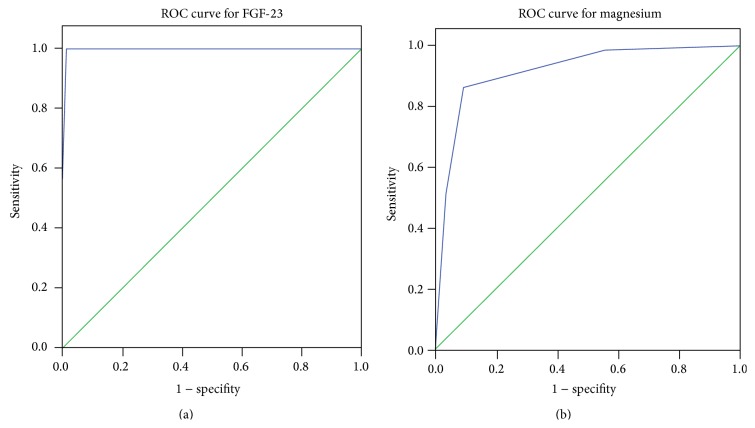
ROC curve analysis results for serum levels of FGF-23 (a) and magnesium (b).

Finally, the cut-off levels of FGF-23 and magnesium were determined by the ROC curve analysis to differentiate between patients with and without mitral valve calcification. The area under the curve for FGF-23 and magnesium was 0.997 ± 0.003, *P* < 0.001 and 0.916 ± 0.024, *P* < 0.001, respectively (Figure 5). The mean cut-off value obtained for FGF-23 was 117 RU/mL and for magnesium the mean cut-off value was 1.7 mg/dL (= 0.7 mmol/L).

## 4. Discussion

As a multifactorial process, many variables are thought to be responsible for vascular and valvular calcification in dialysis patients, including the duration of dialysis, diabetes and inflammation. Recently the role of mineral metabolism deregulation in the pathophysiology of calcification has been gaining relevance [34–36], with several studies associating serum magnesium and FGF-23 levels with increased vascular calcification in dialysis patients [4, 37, 38].

On the other hand the association between low serum magnesium levels and diabetes [39–41], particularly with hypomagnesemia being associated with a higher prevalence of diabetes [42] has also been demonstrated in several studies. However, as far as our knowledge goes, no other study before has assessed the relationship of magnesium and FGF-23 with mitral valve calcification and IMT in diabetic subjects with mild to moderate CKD. To elucidate this question we analyzed 150 diabetic patients with CKD 2–4. After patient allocation to groups according to their Wilkins score almost every variable assessed had shown statistically significant differences between groups. However, in multivariate linear regression only magnesium, FGF-23, calcium, and eGFR were found to be independent predictors of mitral valve calcification. Furthermore, multivariate linear regression regarding IMT revealed once again magnesium and FGF-23 as independent predictors, this time together with IL-6 and the Wilkins score. Taken together the presented data are indicative that these two variables can be considered as predictors of mitral valve calcification and that this condition also alters vascular mechanical properties [4, 42, 43].

Magnesium exerts its protective effect on vascular calcification through multiple molecular mechanisms [4, 15, 23]. In particular it seems to negatively regulate vascular calcification and osteogenic differentiation through transient receptor potential melastatin (TRPM)7 activity and increased expression of anticalcification proteins [44]. Observational data suggest that magnesium may play an important role in the development and acceleration of arterial atherosclerosis and vascular calcification both in patients with CKD and in the general population [4, 42].

Levels of FGF-23 rise early in the course of CKD for that normal phosphorus levels can be maintained [45]. It is thought that these changes may be due to compensatory effects on phosphate retention caused by decreasing capacity of the damaged kidney to excrete dietary phosphorus loads, increased FGF-23 secretion into circulation, and decreased FGF-23 removal from circulation [46]. However, this rise has been associated with worse outcome [47]. Several studies with dialysis and predialysis CKD patients also suggest an association between high FGF-23 levels and vascular calcification [35, 37, 38, 40, 48].

More detailed analyses of our data regarding the role of magnesium on survival analyses suggested that mortality rates were higher both in patients with poorer grades of mitral valve calcification and in the subset of patients with lower magnesium levels. The association between hypomagnesaemia and mortality is further indicated by the strong inversed trend of patient numbers and calcification score in the two magnesium groups (magnesium < 1.85 mg/dL: calcification score 1: *n* = 4; 2: *n* = 10; 3: *n* = 25; 4: *n* = 36 versus ≥1.85 mg/dL: calcification score 1: *n* = 34; 2: *n* = 37; 3: *n* = 4; 4: *n* = 0) and is in accordance with previous studies [11, 13, 14, 49, 50]. Taken together, it seems plausible to assume that magnesium exerts a protective role and that FGF-23 might have a procalcification role in patients not yet undergoing a renal replacement therapy. The correct understanding of these risk factors (hypomagnesaemia and high FGF-23 levels) for mitral valve calcification in predialysis patients is extremely important as the presence and the extent of valvular calcifications impact patient survival.

Thus, magnesium levels might have a significant clinical relevance as a marker or predictor of mitral valve calcification as well as IMT as a measure of VC and last but not least, a therapeutic role for magnesium should be considered. In addition, magnesium and FGF-23 may potentially be used as targets for early interventions in predialysis patients in order to manage these risk factors for calcification, thereby possibly modulating its progression for that cardiovascular mortality might be reduced before dialysis as well as when these patients enter dialysis. In both ways the cut-off levels determined here might be of help for clinical practice.

There are several limitations in the current study such as the relatively small sample size and, consequently, the limited statistical power of the tests applied. Nevertheless, these are the first results of an ongoing long-time project and the main objective of this analysis was to establish primary associations and to put forward further studies to clarify and better understand the role of magnesium and FGF-23 in the pathophysiology of calcification in diabetic CKD patients. Thus, prospective studies with bigger sample size and robust statistical analysis are required in order to confirm these associations.

In conclusion, the present study shows that a deregulation of mineral metabolism, with particular attention directed to magnesium and FGF-23, impacts the extent and severity of mitral valve annulus calcification and IMT on type 2 diabetic patients with a diagnosis of mild to moderate CKD.

## Figures and Tables

**Table 1:** Grading of mitral valve characteristics according to Wilkins score.

Grade	Mobility	Thickening	Calcification	Subvalvular thickening
1	Highly mobile valve with only 1 leaflet tips restricted	Leaflets near normal in thickness (4-5 mm)	A single area of increased echo brightness	A single area of increased echo brightness

2	Leaflet midportions and base portions have normal mobility	Midleaflets normal, considerable thickening of margins (5–8 mm)	Scattered areas of brightness confined to leaflet margins	Scattered areas of brightness confined to leaflet margins

3	Valve continues to move forward in diastole, mainly from the base	Thickening extending through the entire leaflet (5–8 mm)	Brightness extending into the midportion of the leaflets	Thickening extending to the distal third of the chords

4	No or minimal forward movement of the leaflets in diastole	Considerable thickening of all leaflet tissue (>8–10 mm)	Extensive brightness throughout much of the leaflet tissue	Extensive thickening and shortening

**Table 2:** Patients' baseline characteristics.

Parameter	Values
Number of patients enrolled, *n*	150
Age (years)	66.6 ± 9.7
Gender, M/F (%)	97/53 (64.7/35.3)
Hb (g/dL)	13.0 ± 1.5
Blood Pressure (mmHg)	127.2/74.4 ± 8.5/8.1
HbA1c (%)	6.9 ± 0.8
Total cholesterol (mg/dL)	188.8 ± 40.8
HDL (mg/dL)	41.1 ± 10.1
LDL (mg/dL)	106.1 ± 34.4
Triglycerides (mg/dL)	141.6 ± 67.0
Creatinine (mg/dL)	1.7 ± 0.9
eGFR (mL/min)	49.7 ± 21.0
Albumin/creatinine ratio (*μ*g/mg)	111.7 ± 78.7
Magnesium (mg/dL)	1.7 ± 0.7
Phosphorus (mg/dL)	3.7 ± 0.6
PTH (pg/mL)	145.7 ± 92.9
Calcium × phosphorus (mg/dL)	35.1 ± 5.8
FGF-23 (RU/mL)	112.5 ± 66.8
1,25(OH)_2_D3 (pg/mL)	21.1 ± 8.6
IL-6 (pg/mL)	7.3 ± 3.7

**Table 3:** Multivariate linear regression analysis of influencing factors of mitral valve calcification.

Variable	Coefficient	SE	*P* value
Age	0.011	0.006	0.101
Calcium	0.345	0.140	0.029
Phosphorus	0.066	0.158	0.675
Ca × P	0.999	0.018	0.591
PTH	<0.001	0.001	0.517
Creatinine	0.023	0.137	0.869
eGFR	−0.012	0.005	0.026
FGF-23	0.028	0.001	<0.001
Magnesium	−0.916	0.112	<0.001
1,25(OH)_2_D3	−0.011	0.012	0.352
IL-6	<0.001	0.025	0.971

**Table 4:** Multivariate linear regression analysis of influencing factors of IMT.

Variable	Coefficient	SE	*P* value
Age	0.001	0.001	0.483
Calcium	0.004	0.031	0.908
Phosphorus	0.003	0.034	0.935
Ca × P	0.006	0.004	0.146
PTH	<0.001	<0.001	0.463
Creatinine	0.055	0.029	0.066
eGFR	0.001	0.001	0.471
FGF-23	0.001	0.001	0.041
Magnesium	−0.060	0.029	0.002
1,25(OH)_2_D3	0.002	0.003	0.492
IL-6	0.016	0.005	0.005
Wilkins Score	0.073	0.018	<0.001
